# Severe Stress among Medical Students of Two Medical Colleges: A Descriptive Cross-sectional Study

**DOI:** 10.31729/jnma.7196

**Published:** 2022-08-31

**Authors:** Janak Khadka, Pravesh Bhattarai, Shikshya Chapagain, Naresh Manandhar, Rachana Sharma

**Affiliations:** 1 Kathmandu Medical College and Teaching Hospital, Sinamangal, Kathmandu, Nepal; 2 Kathmandu University School of Medical Sciences, Dhulikhel, Kavre, Nepal; 3 Department of Community Medicine, Kathmandu Medical College and Teaching Hospital, Sinamangal, Kathmandu, Nepal; 4 Department of Psychiatry, Kathmandu Medical College and Teaching Hospital, Sinamangal, Kathmandu, Nepal

**Keywords:** *coping strategies*, *medical students*, *psychological stress*

## Abstract

**Introduction:**

Mental health problems are common in medical students and there is a high prevalence of psychological morbidity among them. The inability to use effective coping strategies affects the health and academic performance of students. The aim of this study was to find out the prevalence of severe stress among medical students of two medical colleges.

**Methods:**

A descriptive cross-sectional study was conducted among medical undergraduates of two medical colleges from 14 September to 14 October 2021. Ethical approval was taken from the Institutional Review Committee (Reference number: 0609202109). Three hundred fifteen students were selected for the study using the proportionate stratified random sampling technique. Perceived Stress Scale was used to assess the level of stress and the Brief Cope Inventory scale was used to assess the coping strategies employed by students during stress. Point estimate and 95% confidence interval was calculated.

**Results:**

Among 315 medical students, severe stress was found in 39 (12.38%) (8.76-16.04, 95% Confidence Interval).

**Conclusions:**

The prevalence of severe stress among medical students was higher when compared to similar studies done in similar settings.

## INTRODUCTION

Stress is any physical or psychological stimulus that disturbs the adaptive state and provokes a coping response exceeding the resources of the person.^1^ Coping is the thoughts and behaviours used to manage the internal and external demands of situations that are appraised as stressful.^2^

Mental health problems are common in medical students and there is a high prevalence of depression, anxiety and burnout among medical students.^3^ Psychological morbidity along with major health problems like stroke, heart attack, and obesity are closely related to severe stress.^4,5^ The past study reflected that individuals using adaptive coping strategies had a lower level of stress, and those using maladaptive coping had a very negative impact on health and performance.^6^ Since medical students face a number of stressors during their training, active positive coping is important in the medical career.

The aims of this study was to find the prevalence of severe stress among medical students of two medical colleges.

## METHODS

The descriptive cross-sectional study was conducted among undergraduate medical students studying at Kathmandu Medical College (KMC) and Kathmandu University School of Medical Sciences (KUSMS) from 14 September to 14 October 2021. Ethical approval was obtained from the Institutional Review Committee of Kathmandu medical college and teaching hospital (KMCTH)on 13 September 2021 with (Reference number: 0609202109). The study population consisted of students of Bachelor in Medicine and Bachelor in Surgery (MBBS) and Bachelor in Dental Surgery (BDS) from the first to the fourth year of both tertiary care hospitals. The interns of both faculty were excluded. Those who gave voluntary consent were included in the study. The sample size was calculated using the formula given below:


$$\begin{array}{ll} \text{n} & ={\text{Z}}^{2}\times \frac{\text{p}\times \text{q}}{{\text{e}}^{\text{2}}}\\ & =1.96^{2}\times \frac{0.50\times 0.50}{0.06^{2}}\\ & =267 \end{array}$$

Where,

n= minimum required sample size
Z= 1.96 at 95% Confidence Interval (CI)
p= prevalence taken as 50% for maximum sample size calculation
q=1-p
e= Margin of error, 6%

Thus, the calculated sample size was 267. The total number of students from MBBS and BDS of KMCTH and KUSMS was 1098. The above-calculated sample size is adjusted for a finite population as:


$$\begin{array}{ll} \text{n'} & =\frac{\text{n}}{1+\frac{\text{(n}-1)}{\text{N}}}\\ & =\frac{\text{267}}{1+\frac{\text{(267}-1)}{\text{1098}}}\\ & =215 \end{array}$$

Where

n'= adjusted sample size for a finite population
N= total number of students

Thus, the final sample size was 215. By adding a 10% non-response rate, the sample size was 239. However, final sample size taken was 315.

A total of 1098 students currently enrolled in each batch were taken from the academic record section of the colleges. Participants from the first to the fourth year of MBBS and BDS faculty of both colleges were selected using a proportionate stratified random sampling method. Total students were stratified into sixteen strata, eight strata from KMC, and eight strata from KUSMS. Among eight strata from each college, four strata were taken from MBBS and four strata were taken from BDS faculty. To incorporate students from all batches of both faculty of two colleges each student of each batch was assigned a particular number according to the administrative roll number. Then the study participants were selected randomly using the computer method keeping in mind the proportion of students from each year. Students were contacted personally through social media like Viber, messenger, and email with the help of class representatives of every batch.

Perceived Stress Scale (PSS) was used to assess the level of severe stress of medical students. It has good reliability and procedural validity.^7^ It consists of 10 items rated on 5 points Likert scale (0: never, 1: infrequently, 2: sometimes, 3: frequently, 4: always). Scores ranging from 0-13 were considered mild stress, 14-26 as moderate stress and 27-40 were considered severe stress.

Coping strategies adopted by students to deal with stress were measured by using valid and reliable tools i.e. Brief Cope Inventory (BCI) scale.^8^ It consists of 14 scales each having 2 subscales, thus a total of 28 items.

Data were entered and analysed using IBM SPSS version 21.0. Point estimate and 95% CI was calculated.

## RESULTS

Among 315 medical students of two medical colleges, the prevalence of Severe Stress was 39 (12.38%) (8.76-16.4, 95% Confidence Interval). Out of the students with severe stress, the higher number of students 7 (17.95%) were from BDS first year and the lower number of students 2 (5.12%) were from MBBS third year (Table 1).

**Table 1:** Descriptive analysis of severe stress among medical students of two medical colleges (n= 39).

Year of study	MBBS n (%)	BDS n (%)
First-year	6 (15.38)	7 (17.95)
Second-year	4 (10.26)	6 (15.38)
Third-year	2 (5.13)	6 (15.38)
Fourth-year	5 (12.82)	3 (7.69)

Of the 39 severely stressed students, 8 (20.51%) were male and 31 (79.49%) were female. The majority of students with severe stress were seen in the age group 21-22 of 18 (46.15%). Severe stress according to faculty was found to be 22 (56.41%) in BDS and 17 (43.59%) in MBBS (Table 2).

**Table 2:** Demographic profile of the participants (n= 39).

Demographic		n (%)
Age	19-20	12 (30.77)
	21-22	18 (46.15)
	23-24	9 (23.08)
Sex	Male	8 (20.51)
	Female	31 (79.49)
Tertiary care hospitals	KUSMS	23 (58.97)
	KMC	16 (41.03)
Faculty	BDS	22 (56.41)
	MBBS	17 (43.59)
Living conditions	Hostel	12 (30.77)
	Rented house	12 (30.77)
	With family	12 (30.77)
	With friends	3 (7.69)
Parent occupation	Business	10 (25.64)
	Army	3 (7.69)
	Engineer	2 (5.13)
	Lecturer	2 (5.13)
	Others	22 (56.41)

The different variables of coping measured by BCI revealed that self-blame followed by acceptance and planning were the most commonly used coping strategies by medical students with severe stress. This study depicted that substance use, humour and religion were the least three common coping strategies employed by severely stressed medical students (Table 3).

**Table 3:** Descriptive statistics for coping strategies used by medical students of two medical colleges having severe stress (n= 39).

Coping strategy	Mean±SD
Self-blame	3.23±0.85
Acceptance	2.77±0.58
Planning	2.67±0.70
Venting	2.64±0.78
Self-distraction	2.56±0.81
Active coping	2.54±0.78
Positive reframing	2.45±0.85
Behavioral disengagement	2.28±0.82
Using emotional support	2.20±0.94
Using instrumental support	2.19±0.99
Denial	2.13±0.91
Religion	1.91±0.94
Humor	1.85±0.89
Substance use	1.17±0.45

## DISCUSSION

Medical school is a stressful period of physical training. Medical students experience substantial distress during their medical journey.^9^ They face multifactorial stressors like academic (course length, reading lots of textbooks, understanding the vast course, examination) emotional, physical, familial, and social stressors.^10^ Psychological morbidity is closely related to stress.^4^ Studies showed that active coping, planning, acceptance, and positive reframing are protective factors of depression and anxiety.^11^

In our study, the prevalence of severe stress (12.38%) among medical students was found to be increased in comparison to studies done in Kathmandu and Mysore which found the prevalence of severe stress at 6.13% and 6% respectively.^12,13^ This might be because those studies were conducted before the COVID-19 pandemic. A study in Dublin revealed that students' severe stress levels increased after the COVID-19 pandemic.^14^ Among medical students of both colleges, moderate stress (71.42%) was found in the majority of students which concurs with the study done in Mysore which found maximum students have moderate stress.^13^

This study found that severe stress level is related to the academic year of students. The severe stress decreased with the increase in the academic year. This might be due to the psychiatric class and posting in third and fourth-year students. Moreover, it might be due to the fact that skills and experience to deal with stressors developed with the progress of higher study and more exposure. A past study done among medical students of Patan Academy of Health Sciences supported the findings of decreased severe stress with an increase in the academic year.^15^ However, the severe stress of final year MBBS students was high. The findings concur with the study done in Saudi Arabia.^16^ The reason can be the vastness of the academic curriculum in the final year.

Our study also found that the level of severe stress is low in third-year MBBS students (25%). The duration of the third year is of 18 months which is longer in comparison to the duration of another year which is of 12 months. This can be due to the adequate time for preparations for the exam. A similar study done in the Patan Academy of Health Sciences depicted that severe stress increased with less time to study for exams.^15^ A Study in India contradicted our result, where severe stress was found to be more in second and third-year MBBS rather than first year.^17^ One possible reason for these findings can be a difference in the curriculum of the study population.

Self-blame is the most commonly used coping strategy to combat severe distress.^18^ This finding was similar to our result. Acceptance and planning were also commonly used coping strategies after self-blame by medical students to combat severe stress supported by a study done in Pokhara.^19^ However, some study findings contraindicated with findings of our study of where most students adopted substance use, using emotional support from others, and religions as coping strategies. Maladaptive coping is an ineffective way of coping with distressful situations. By employing them, individuals will not succeed in real problems, and it is associated with an increase in alcohol use problems.^20^ Students who engaged in negative coping had a high severe stress level, whereas students using positive coping had a low severe stress level.^14^

The number of students using negative coping is also in significant number. So, early intervention should have to be done by families, colleges, and stakeholders. Health promotion programs in medical schools in the United States reported a reduced result of the negative effects of stress on the health and academic performance of medical students.^21^ Our findings can give the medical colleges and stakeholders feedback to include primary preventive measures such as seminars on stress management and positive coping techniques at the time of admission and basic level along with clinical levels.

The present study was conducted in two medical colleges; hence the findings cannot be generalized among the medical students of Nepal. The validity and reliability of the BCI scale were still not measured in the Nepalese population. So, the results may reflect a certain level of bias. As the study did not cover students' stressors, studies focusing on different stressors need to be carried out.

## CONCLUSIONS

The prevalence of severe stress among medical students was higher than the studies done in similar settings. Effective preventive measures like awareness about the manifestation of distress, counselling on stress management, along with positive coping strategies by professional counsellors should be increased in medical colleges to avoid severe consequences for medical students.
